# A Multi‐Step Model of Parkinson's Disease Pathogenesis

**DOI:** 10.1002/mds.28719

**Published:** 2021-08-10

**Authors:** Campbell Le Heron, Michael MacAskill, Deborah Mason, John Dalrymple‐Alford, Tim Anderson, Toni Pitcher, Daniel Myall

**Affiliations:** ^1^ New Zealand Brain Research Institute Christchurch New Zealand; ^2^ Department of Neurology Canterbury District Health Board Christchurch New Zealand; ^3^ Department of Medicine University of Otago Christchurch New Zealand; ^4^ School of Psychology, Speech and Hearing University of Canterbury Christchurch New Zealand; ^5^ Brain Research New Zealand Rangahau Roro Aotearoa Dunedin New Zealand

**Keywords:** incidence, modeling, multistep, Parkinson's disease, pathogenesis

## Abstract

**Background:**

Parkinson's disease (PD) may result from the combined effect of multiple etiological factors. The relationship between disease incidence and age, as demonstrated in the cancer literature, can be used to model a multistep pathogenic process, potentially affording unique insights into disease development.

**Objectives:**

We tested whether the observed incidence of PD is consistent with a multistep process, estimated the number of steps required and whether this varies with age, and examined drivers of sex differences in PD incidence.

**Methods:**

Our validated probabilistic modeling process, based on medication prescribing, generated nationwide age‐ and sex‐adjusted PD incidence data spanning 2006–2017. Models of log(incidence) versus log(age) were compared using Bayes factors, to estimate (1) if a linear relationship was present (indicative of a multistep process); (2) the relationship's slope (one less than number of steps); (3) whether slope was lower at younger ages; and (4) whether slope or *y*‐intercept varied with sex.

**Results:**

Across >15,000 incident cases of PD, there was a clear linear relationship between log(age) and log(incidence). Evidence was strongest for a model with an initial slope of 5.2 [3.8, 6.4], an inflexion point at age 45, and beyond this a slope of 6.8 [6.4, 7.2]. There was evidence for the intercept varying by sex, but no evidence for slope being sex‐dependent.

**Conclusions:**

The age‐specific incidence of PD is consistent with a process that develops in multiple, discrete steps – on average six before age 45 and eight after. The model supports theories emphasizing the primacy of environmental factors in driving sex differences in PD incidence. © 2021 The Authors. *Movement Disorders* published by Wiley Periodicals LLC on behalf of International Parkinson and Movement Disorder Society

Parkinson's disease (PD) is a common neurodegenerative disorder across the world.^1^, ^2^ Its burgeoning prevalence and associated societal impact makes developing effective preventative therapies a global health issue, but incomplete understanding of PD pathogenesis limits this goal.^3^, ^4^ Over time, many mechanisms for disease development have been posited, including genetic, inflammatory, infectious, and other environmental exposures.^3^, ^4^, ^5^, ^6^, ^7^, ^8^, ^9^ Most current models of pathogenesis acknowledge the likelihood that, in most people, the development of the pathological changes that define PD is the final product of a complex interaction between many of these potential etiological factors. Although these observations suggest that PD might develop as a multistep process, empirical evidence for such an overarching framework is lacking, as are specific details such as the number of pathogenic steps required to cause the disease.

The application of techniques developed to understand another common disorder—cancer—to the field of neurodegeneration shows promise for elucidating disease mechanisms.^10^ In particular, a strong body of research demonstrates how epidemiological data can be used to infer the underlying pattern of cancer development, and whether a multistep process is likely to be present.^11^, ^12^, ^13^ Specifically, Armitage and Doll demonstrated that if the onset of a disease requires multiple prior steps, each with a relatively low probability of occurring per year, then a power relationship will exist between the incidence of the disease and age (Box 1 and see Webster 2019 for a full derivation).^13^ Furthermore, the exponent in this power relationship (ie, the slope of the log–log line) is one less than the total number of steps required to cause the disease.^11^ This insight has significantly enhanced the understanding of cancer biology.^11^, ^12^, ^14^ Furthermore, the potential utility of this approach for understanding the pathogenesis of neurodegenerative conditions has been demonstrated in amyotrophic lateral sclerosis (ALS), where the incidence rate was found to increase with age in a manner predicted by a multistage process requiring six events for disease development.^15^ Additionally, the contribution of single monogenic “causes” of ALS reduces the required number of steps to between two and five depending on the mutation.^16^

Box 1 Armitage and Doll Multistep Model – Heuristic Argument*

If disease development depends on one step, incidence in a given year will be proportional to the chance of undergoing that step:

$$I\left(t\right)\propto k$$

If two‐steps are required, then incidence is the product of the chance of undergoing the first step by age *t* and the rate of undergoing the second step:

$$I\left(t\right)\propto k_{1}t\times k_{2}$$

And for n‐steps:

$$I\left(t\right)\propto k_{1}t\times k_{2}t\;\ldots \;\times k_{n-1}t\times k_{n}$$

$$\text{Or}:\;I\left(t\right)\propto k_{1}k_{2}\;\ldots \;k_{n-1}k_{n}t^{n-1}$$

Taking log of both sides returns the equation for a straight line:

$$\log\left(I\left(t\right)\right)=\left(n-1\right)\;\log\left(t\right)+c$$

The slope is one less than the number of steps required to develop the disease, and the intercept represents the combined probability of undergoing these steps.

*see Webster 2019 for full derivation.^13^

A multistep model of pathogenesis could account for many of the epidemiological observations made in PD. These include the variability in the expression of disease and age of onset in carriers of disease‐causing mutations,^6^, ^17^, ^18^ and the multiple environmental and genetic associations that confer a risk of developing PD.^1^, ^3^, ^6^ A multistep model could also explain the phenotypic variability seen in PD, if it is assumed that at least some steps apply to specific neuronal populations rather than the nervous system as a whole.^8^ Furthermore, the predictions arising from such a model can be used to test specific hypotheses about basic observations in PD, such as whether the higher incidence and prevalence of PD in males observed in most parts of the world relates to differential environmental exposures by sex.^2^

One significant hurdle to investigating the validity of this model of PD pathogenesis has been the difficulty in acquiring large‐scale, reliable incidence data. Recently, we applied a Bayesian model to national‐level drug prescribing data, validated on diagnostic information within a subgroup of the population, to estimate age‐standardized PD incidence for the whole of New Zealand (a country of approximately 5 million people) across a 10‐year period.^19^, ^20^ Here, we use this data set—extended to include 12 years of nationwide age‐standardized PD incidence values—and the Armitage‐Doll model, to test the hypothesis that Parkinson's disease pathogenesis is a multistep process, and to determine the number of steps required for disease development.

We then use this multistep framework to ask three specific questions. First, is the incidence data better explained by two separate groups with different slopes? This would be expected if those with younger onset had developed PD under stronger genetic influence, thus requiring fewer additional steps. Second, do the log incidence curves for males and females differ in a manner consistent with varying environmental exposure effects or fundamentally differing pathogenic pathways? Finally, the incidence of PD has been observed to plateau and eventually decline in very elderly people.^19^, ^21^ We therefore investigate whether extensions of the Armitage‐Doll model, also derived from the cancer literature,^22^, ^23^ can explain this phenomenon.

## Patient and Methods

**FIG. 1:**
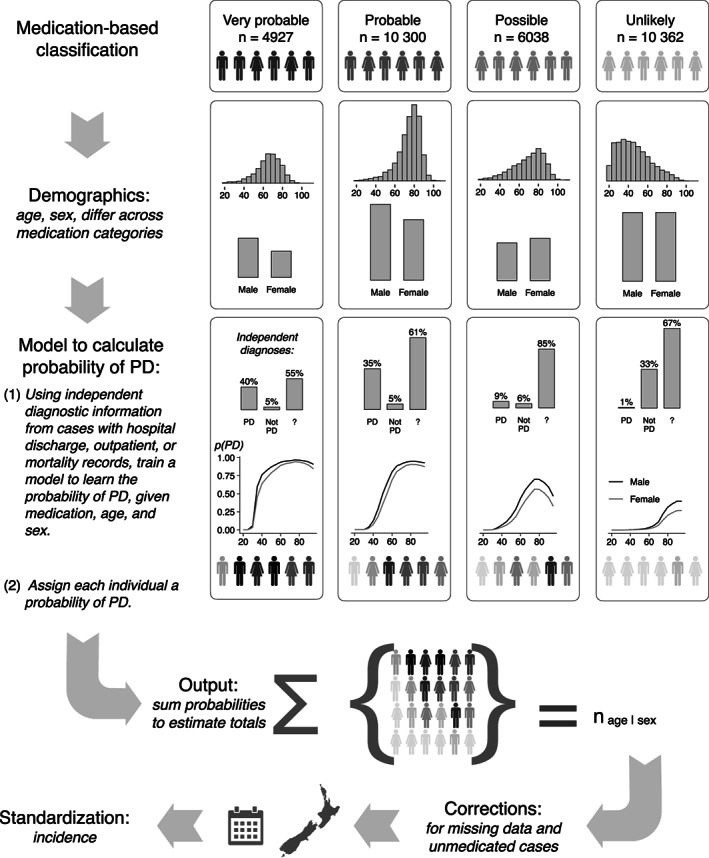
The probabilistic modeling process. Potential Parkinson's disease (PD) cases were identified by our medication‐based classification. Within each classification category (“very probable” through “unlikely”), individual cases started with the same (initially unquantified) probability of having Parkinson's, as indicated by the common shading of the silhouettes. The demographic characteristics were then quantified. For example, the “very probable” and “probable” categories showed age distributions and sex ratios consistent with those expected for a PD population. By contrast, the “unlikely” category, expected to be dominated by anticholinergic use for psychiatric purposes, was skewed toward younger cases, with a more equal male:female ratio. We then sought independent diagnostic information from other data sources, allowing us to confirm “PD” or some other condition (“not PD”) in a subset of cases. In the remaining cases, the diagnosis remained unknown (“?”). This information was used to train a model to learn the probability of a case having Parkinson's, given the patient's medication use, age, and sex. Each case was assigned such a probability (as indicated by the now variously shaded silhouettes). Therefore, our estimated total numbers of people with PD in each age and sex grouping are not counts of discrete, identified individuals. Rather, they are formed by summing up the continuous probabilities assigned to individuals. These totals were then standardized by the census‐derived national age, and sex distributions and each case's period of occurrence within the data set to form estimates of age and age‐sex‐specific incidence. Figure by Myall, Le Heron, MacAskill (2021), distributed at https://doi.org/10.6084/m9.figshare.13934855 under a CC‐BY licence.

The age‐specific incidence of Parkinson's for people over the age of 30 was calculated over the years 2006–2017 for the entire New Zealand population using previously described methods (Fig. 1).^19^, ^20^ Briefly, all individuals in the country who took anti‐parkinsonian medications were identified. For a subset of these, a Parkinson's or non‐Parkinson's diagnosis was determined from all available clinical data (neurologist reviews, hospital records, Parkinson's society databases). This allowed us to determine the probability of each individual having Parkinson's given the type and dose of medications they took over time, and their age and sex. By summing up these probabilities for all individuals by each year, sex, and age grouping we could determine the national age‐sex‐specific incidence by year. The accuracy of model calibration was further checked in a subset of people who had been reviewed by a movement disorders neurologist (TA).^19^, ^20^

Bayesian regression models defined in Stan and fit in RStan (https://mc-stan.org/users/interfaces/rstan) were used to determine the model parameters (slopes, intercepts, inflexion points). The median of the posterior distribution is given as the parameter estimate, along with credible intervals defined by 2.5% and 97.5% percentile quantiles from the posterior distribution. Full data, model specification, and analysis code are available at https://github.com/nzbri/parkinsons-multistep.

The Armitage–Doll model is:

$$I\left(t\right)=\alpha t^{k-1}$$

where $I\left(t\right)$ is incidence, t is age, α is the product of the exposure risks for each step, and k is the number of slow stages (steps). Taking the log of both sides gives the linear equation:

$$\log\left(I\left(t\right)\right)=\left(k-1\right)\log\left(t\right)+c$$

where $c=\left(k-1\right)\log\left(\alpha \right)$.

The basic Armitage–Doll model cannot account for a reduction in incidence at very old ages (>80 years for Parkinson's), as is observed in most cancers and in Parkinson's.^19^, ^22^, ^23^ We therefore restricted fitting the basic Armitage–Doll model to a maximum of age 80 years, based on the visual inspection of the incidence data and previous approaches.^15^

A linear regression, taking into account the uncertainty in the age‐specific incidence, was used to examine the fit of the standard Armitage–Doll model. A Bayesian R‐squared measure, based on the median age‐specific values, was used to determine how well the data points fitted the model.^24^ Then, to test the hypothesis that the data set contained two separate populations with differing number of steps required to develop Parkinson's, we extended the Bayesian regression model to a broken‐stick model to allow an unknown breakpoint $t_{\mathrm{bp}}$ after which the slope increased:

$$\log\left(I\left(t\right)\right)=\left\{\begin{array}{l} \left(k_{1}-1\right)\log\left(t\right)+c_{1}\;\text{for}\;t<=t_{\mathrm{bp}}\\ \left(k_{2}-1\right)\log\left(t\right)+c_{2}\;\text{for}\;t>t_{\mathrm{bp}} \end{array}\right.$$

where $c_{i}=\left(k_{i}-1\right)\log\left(\alpha_{i}\right)$ for i∈1,2

Finally, to test for sex differences, the regression model was further extended to allow both the intercept and slope (but not breakpoint) to vary by sex. Bayes factors were used to determine if the extended models provided a better fit of the data. Briefly, Bayes factors give the ratio of the likelihood of the data given one potential model to the likelihood of the data given a second, alternative, model. Conventionally, the degree of evidence for a particular model is considered moderate (3–10), strong (10–100), or decisive (>100).^25^ Informative priors, based on estimated parameter values from prior applications of the Armitage‐Doll model and the scale of the observed data, were used in the models. These informative priors limited the scale of plausible parameters to moderately tight intervals and were used to allow the calculation of Bayes factor values and to improve nonlinear model convergence.

In the cancer literature, as in our data, the linear Armitage–Doll relationship holds only until a certain age, after which incidence tends to reduce markedly. Two prominent extensions of the Armitage‐Doll model, the *beta model* and the *susceptibility model*, have been applied to account for this. Simplistically, these models can be considered to relate to factors occurring at each end of the pathogenic process.

The beta model^22^ has the form:

$$I\left(t\right)=\alpha t^{k-1}\left(1-\mathrm{\beta t}\right)$$

where the additional term 1−βt represents a disease “extinction” factor, which could, for example, represent cells becoming deactivated rather than spreading the pathology. Thus, it has the effect of increasingly reducing disease incidence as age (and accumulation of pathogenic “hits”) increases.^12^ An analogous situation could arise in PD pathogenesis, for example, related to disrupted metabolic cell dynamics.^5^

In contrast, the susceptibility model^23^, ^26^ has the form:

$$I\left(t\right)=\frac{\alpha t^{k-1}}{1+\frac{1-C}{C}\exp\left(\frac{\alpha }{k}\left(t^{k}-1\right)\right)}$$

where C is the initial proportion of the population that is “susceptible” to the disease and 1−C is the proportion that is immune. Thus, it predicts decreasing disease incidence at high ages because the initial pool of people who could ever develop disease becomes depleted over time. Three parameters are estimated in this model—the initial proportion of susceptible individuals within a population $\left(C\right)$, the steps required to develop PD $\left(k\right)$, and the combined risk of exposure to these steps $\left(\alpha \right)$.

We applied each of these extension models (neither of which includes a change‐point parameter) to the full data set (age range 30–100+) and compared their fit to the basic Armitage‐Doll model using Bayes factors.

## Results

**FIG. 2:**
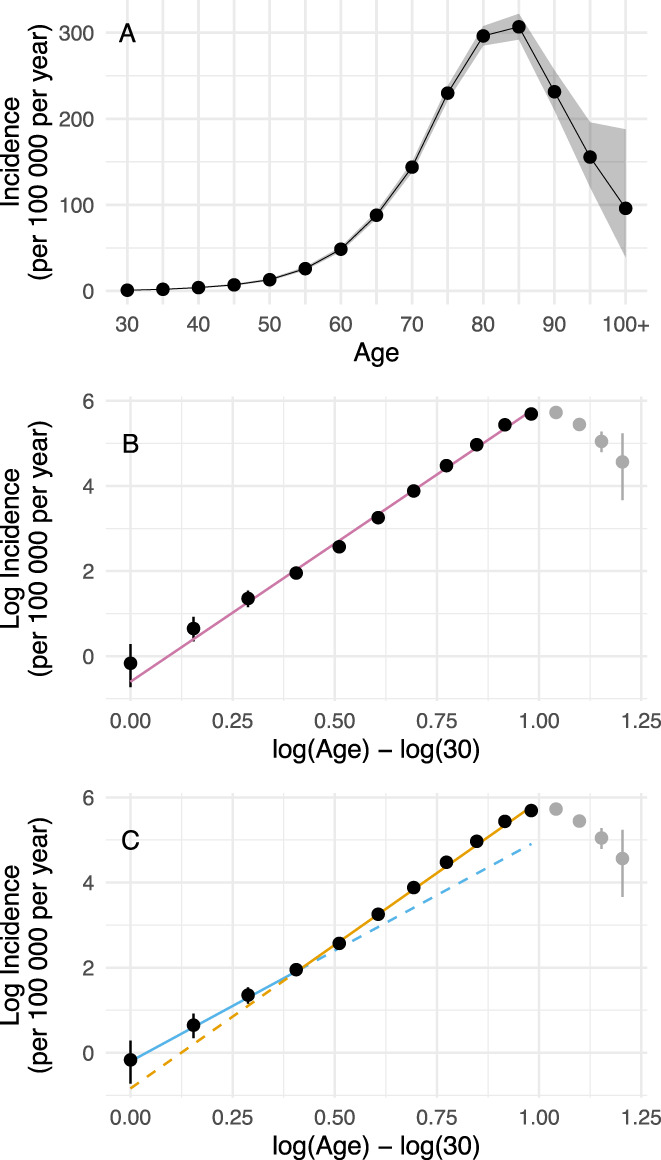
Relationship between log(age) and log(incidence). (**A**) Age‐specific incidence of Parkinson's disease in New Zealand from 2006 to 2017, with the shaded areas representing 95% credible intervals (**B**) The relationship between natural log incidence and log age, with a linear model fit spanning age from 30 to 80. Points not included in the model fitting process are shaded grey. The error bars represent 95% credible intervals of the transformed values. Log (30) is subtracted from log(age) for modeling purposes. (**C**) A broken‐stick regression of the same log‐transformed data, showing a better fit, and suggesting a lower number of steps required for younger‐onset cases (six steps) compared to older cases (eight steps). The regression lines are extended as dashed lines beyond the point of inflection to illustrate the difference in slopes (5.2 until age 45, 6.8 thereafter). Figure by Myall, Le Heron, MacAskill (2021), distributed at https://doi.org/10.6084/m9.figshare.13933274 under a CC‐BY licence. [Color figure can be viewed at wileyonlinelibrary.com]

Over the 12‐year period between 2006 and 2017 we estimated there were 15,500 incident cases of PD in New Zealand. The overall age‐specific incidence is shown in Figure 2A. There is a pattern of an exponential increase until ~80 years, followed by a plateau and then a steady decrease.

### Modeling Power Law Relationship

Working with the Armitage–Doll model, a linear regression model of the log age versus the log incidence until age 80 gave a very good fit of the data (R^2^ = 0.994), with a slope (corresponding to one less than the number of steps required to develop PD) of 6.5 [6.2, 6.7], (Fig. 2B). This value, being intermediate between two integers, suggested that the estimated number of steps required may have arisen from the mixture of more than one sub‐population, and/or that the number changed as a function of age.

### Constant or Changing Slope

To test for a changing slope, a broken‐stick model was evaluated (Fig. 2C). There was strong evidence for this over the simple linear model (Bayes factor = 12), with an estimated initial slope of 5.2 [3.8, 6.4] up until a breakpoint estimated at $t_{\mathrm{bp}}$ = log(age) – log (30) = 0.41 [0.22, 0.65] (corresponding to an age of 45 [37, 57]), beyond which the slope was 6.8 [6.4, 7.2]. This is most consistent with a multistep model where there are a mean of six steps required for the development of the disease before age 45, and 8 steps after this age. Based on the 95% credible intervals, the results would also be consistent with either a five‐ or seven‐step process *before* age 45, although each of these possibilities is less likely than a six‐step process.

### Modeling of Sex

**FIG. 3:**
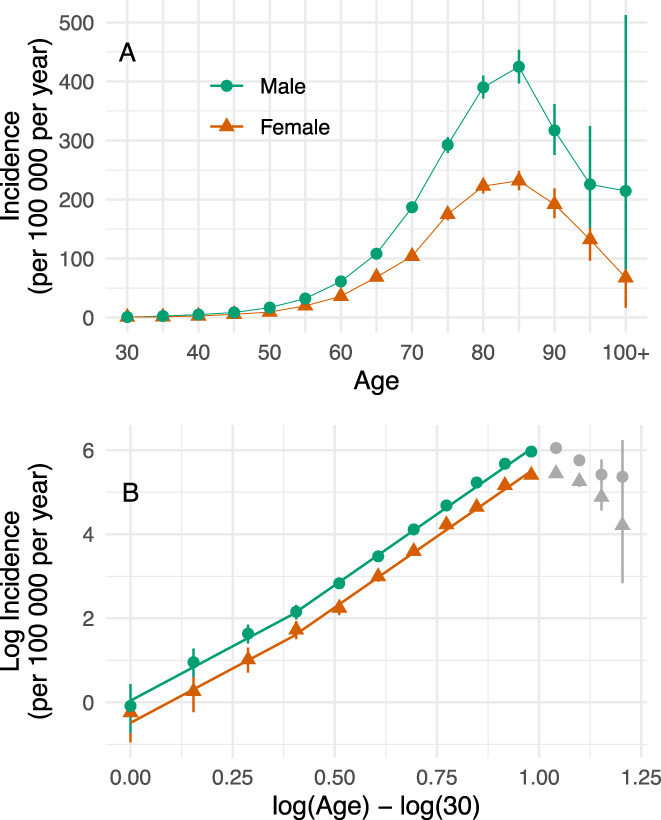
Effect of sex on log(age)/log(incidence) relationship. (**A**) Age‐sex‐specific incidence of Parkinson's disease in New Zealand from 2006 to 2017, with the error bars representing 95% credible intervals. (**B**) The relationship between log incidence and log age when split by sex. Broken‐stick regression models were fit by sex, with evidence for males and females having a difference in intercept, but no evidence for a difference in slopes. Figure by Myall, Le Heron, MacAskill (2021), distributed at https://doi.org/10.6084/m9.figshare.13933835 under a CC‐BY licence. [Color figure can be viewed at wileyonlinelibrary.com]

Standardizing to the overall population age‐distribution, the mean age of onset was 71.5 [71.2, 71.8] for females and 71.7 [71.5, 71.8] for males. When modeling the log relationship, there was extremely strong support for a model that allowed the intercept to vary by sex (Bayes factor = 2 × 10^8^), but no support for a model that also allowed the slope to vary (Bayes factor = 0.04, providing support for the simpler model). The intercept for females was −0.5 [−0.9, −0.1] and for males was 0.0 [−0.3, 0.4]. The resulting broken‐stick regression model with a varying intercept by sex is shown in Figure 3B.

### Modeling the Incidence at Older Ages

**FIG. 4:**
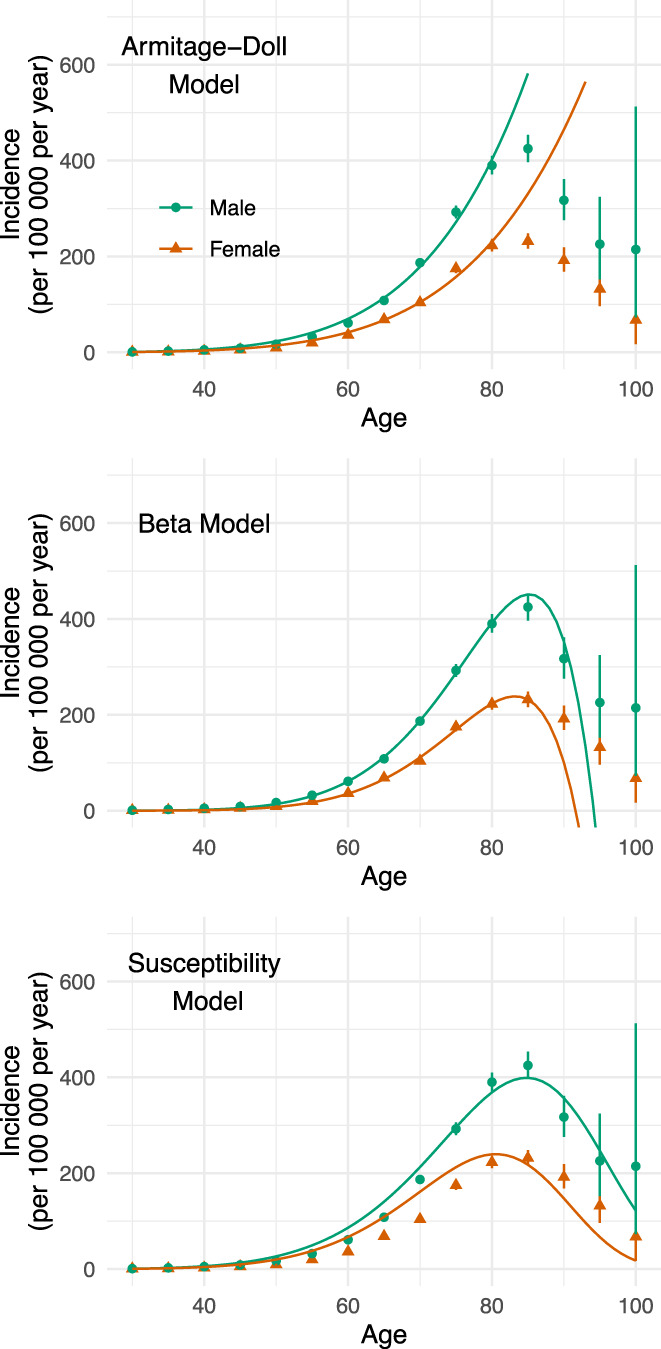
Extended models to capture incidence drop‐off at very old ages. Comparison of models when examining the entire age range. The standard Armitage‐Doll model diverges rapidly from the observed data at the older ages. The beta model supports a drop‐off at older ages, although it cannot fit the observed curvature of the drop‐off, with the biggest limitation of the model being that it predicts that incidence becomes negative beyond age 90. In contrast, the susceptibility model provides a closer match to the data, with predicted incidence values that remain greater than or equal to zero. Figure by Myall, Le Heron, MacAskill (2021), distributed at https://doi.org/10.6084/m9.figshare.13934489 under a CC‐BY licence. [Color figure can be viewed at wileyonlinelibrary.com]

As expected, the classic Armitage–Doll model clearly diverged from the data at elderly ages (>80) (Fig. 4A). In contrast, the beta model (Fig. 4B) could account for some of this reduction in incidence at older ages, although it did not provide a close fit to the data. However, the susceptibility model (Fig. 4C) provided a much closer fit (Bayes factor = 1 × 10^43^ over the beta model). This model predicted that 6% and 11% of the female and male populations, respectively, are initially susceptible to ever developing Parkinson's disease (C_female_ = 6.4% [6.1, 6.6], C_male_ = 11.2% [11.0, 11.5]). Furthermore for both sexes, in those who do develop it, on average eight steps are required (k_female_ = 8.0 [7.6, 8.4], k_male_ = 7.7 [7.5, 7.9]—see Table S1 for all parameter estimates).

## Discussion

Conceptualizations of PD pathogenesis acknowledge the likelihood of a complex interplay between genetic and environmental factors over a person's lifespan.^3^ Beyond this, however, postulates remain sparse yet diverse, with possibilities ranging from a single environmental exposure triggering a slowly evolving disorder, to a far more complex process involving multiple exposures across time.^1^, ^7^, ^27^ The Armitage‐Doll model, originally developed to understand cancer epidemiology and development, provides a mathematical framework to probe this issue. Here, applying this model to a large‐scale incidence data set, we obtained results that are clearly consistent with the hypothesis that PD develops as a multistep process, the last step of which leads to the clinical entity we recognize as PD. In particular, for the vast majority of people who develop PD from middle‐age onward, the model suggests an average of eight events—or steps—are required (Fig. 2C). Furthermore, this model casts new light on the widely observed sex‐difference in PD incidence, with results supporting the notion that the preponderance in males is related to differences in exposure dynamics rather than fundamental pathogenic mechanisms (Fig. 3B). Finally, extensions of this model suggest that it might be early life features, which determine a pool of people susceptible to ever developing PD, that explain the drop‐off in PD incidence observed at very old (>80) age (Fig. 4).

The New Zealand incidence data was fitted reasonably well by a model that assumed a PD population who require the same number of steps to develop disease. However, *a priori* we expected that people who develop PD at a younger age are more likely to have a stronger genetic predisposition,^5^, ^27^, ^28^ and, within a multistep framework, may therefore require fewer additional “hits” to develop disease. Such a relationship has been demonstrated in ALS.^16^ Consistent with this notion, we found stronger evidence for a model that included a mix of two subpopulations, each with differing numbers of steps required. In the best‐fitting model, the slope of the log–log curve was lower at younger ages, with a transition point at approximately 45 years of age. This means that those younger than 45 on average required six steps to develop PD, whereas for those over 45 years (the majority of people), eight steps were required. This extension of the basic model also provides further indirect evidence for the general multistep framework, as it is difficult to explain such a change in log–log curve slope otherwise. It also points to the importance of studying carriers of causative genetic mutations to better understand environmental factors that mediate disease development, and to explore whether particular mutations may be associated with varying numbers of required additional steps.

With just a few exceptions, a consistent epidemiological finding across many populations has been the greater incidence of PD in males compared to females,^2^ an observation also evident in the current study. An open question is whether this difference relates to different environmental exposure effects between sexes. Within the multistep framework, lower exposures to risk factors for PD should reduce the chance of undergoing a given step, without altering the number of steps required to develop PD. Thus, we predicted that a systematic difference in exposures between males and females would alter the intercept term of the model, without changing the slope of the log(age) versus log(incidence) function. Consistent with this, we indeed found no difference in slope between males and females, and a significantly lower intercept term for females. Together, this is consistent with the notion that the PD sex difference is not caused by fundamental differences in disease pathogenesis, but rather by differences in environmental exposures. It is important to note that this data would also be consistent with a biological difference between males and females that, for example, modulates the effects of a given environmental exposure. However, the fact that the PD sex difference is not present in all countries^29^, ^30^, ^31^ would tend to argue against this latter point. These findings also highlight the importance of studying differences between populations that do and do not show a sex difference, to identify specific environmental exposures that lead to PD.

Although a linear log(age)/log(incidence) relationship was clearly present until approximately age 80 years, above this age incidence fell steeply, as observed in previous studies.^2^, ^32^, ^33^ It is unlikely that disease‐independent factors (eg, co‐morbidities or reduced diagnosis rates) alone could account for such a substantial drop‐off. Importantly, similar decreases in incidence at older ages have been observed in many cancer types, leading to modifications of the basic Armitage‐Doll model.^12^ These extended models take two main forms, emphasizing factors that either become increasingly important as age increases or occur earlier in life. Although both models to some extent captured the decline in incidence of PD at the oldest ages, the susceptibility model provided a much better fit to this drop‐off, and aligned with the earlier estimate of eight steps for both females and males. This model suggests that only a proportion of the population is at risk for ever developing PD, and that this proportion varies by sex: 6% of females and 11% of males. If correct, this observation would suggest that, in addition to sex‐related differences in exposures modulating the chance of a given step occurring, there is either an early life process, genetic effect, or both, which determines whether PD can ever develop in an individual, and which varies between men and women. We would, however, suggest caution in over‐interpreting this result. Visual inspection of the susceptibility model fit demonstrates that, although it captured the drop‐off in incidence at old age, it provided an imperfect fit at other time points. Furthermore, the model classifies susceptibility as a binary state, whereas susceptibility to ever developing PD may exist on a spectrum, for example, modulated by the influence of many genetic polymorphisms, somatic mosaicism, or infection exposure.^6^, ^7^, ^34^ A model incorporating such a weighted risk is significantly more complex, potentially less interpretable, and beyond the scope of this current work, but represents an important and evolving avenue for future research.

A multistep framework for PD development has a number of broader implications for existing and future PD research. Clearly, identifying these steps, which may occur widely across the nervous system or more locally, and understanding the relationships between them is a crucial goal.^8^ Variations in the cellular populations where these steps occur may underlie the wide phenotypic variability observed in PD. There may be redundancy within the pathway as well, such that particular steps could occur by differing mechanisms. In such a way evidence for the early role of olfactory and gut dysfunction in PD pathogenesis may be united within this broad multistep framework.^7^, ^9^, ^35^ The framework also explains the wide variability in penetrance and phenotype associated with different causative genetic mutations, as additional steps are still required even in the presence of such changes—further work should explore the multistep model in specific genetic populations (as has been done in ALS)^16^ as well as focus on environmental modulators of additional steps within these groups. A strength of this study is the use of nation‐wide incidence data derived from a robust, validated methodology.^19^, ^20^ However, demonstrating that these results are reproducible in a different population will be an important step in validation, as well as exploring geographical and ethnic influences on the multistep model. Finally, there are many similarities with the current work in PD and findings that have recently been published in ALS.^15^, ^36^, ^37^ This raises the possibility the framework may be applicable to other neurodegenerative disorders.

Some limitations should be considered when interpreting this study. The Armitage‐Doll model assumes that exposures leading to each step are rare and constant throughout life. Although some steps may be necessary before subsequent ones can occur, mathematically this does not change the predicted log–log linear relationship between age and incidence. Thus the model does not distinguish a sequential process from one that could occur in any order.^11^, ^13^ Furthermore, these results apply at a population level and cannot be used to inform us about how many steps an individual patient with PD has undergone. It is also worth noting that although a linear relationship between log(age) and log(incidence) is consistent with a multistep model, it is not specific for it, and other factors could also explain such an observation.^13^ Finally, the model estimates the number of steps leading to the development of PD, but does not necessarily relate to subsequent modifiers of ongoing neurodegeneration once disease onset has occurred.^38^

PD has a massive global impact on health, but despite the identification of many environmental, genetic, and cellular factors associated with disease development, its pathogenesis remains poorly understood. Here, we present empirical evidence that is consistent with a multistep model of disease development. Such a model provides novel insights into common disease observations including the male preponderance of PD, variability in penetrance of disease‐causing genetic mutations, and the substantial drop‐off in incidence at very old ages. The multistep model may form a unifying framework within which ongoing research on pathogenesis can be understood, and which ultimately may lead to strategies to prevent or delay the development of PD.

## Author Roles

1. Research project: A. Conception, B. Organization, C. Execution;

2. Statistical analysis: A. Design, B. Execution, C. Review and critique;

3. Manuscript preparation: A. Writing of the first draft, B. Review and critique.

C.L.H.: 1A, 1B, 1C, 2A, 2C, 3A, 3B

M.M.A.: 1A, 1B, 1C, 2A, 2C, 3B

D.M.: 3B

J.D.‐A.: 3B

T.A.: 3B

T.P.: 3B

D.M.: 1A, 1B, 1C, 2A, 2B, 2C, 3B

## Financial Disclosures for the Past 12 Months

Campbell Le Heron is employed by the Canterbury District Health Board. He has received funding from Canterbury Medical Research Foundation. Michael R. MacAskill is employed by the University of Otago. He received research support from the Canterbury Medical Research Foundation, Neurological Foundation of New Zealand, and the New Zealand Brain Research Institute over the past 12 months. Deborah Mason is employed by the Canterbury District Health Board. She has received payment for advisory meetings and travel grants from both Biogen and Roche. John C. Dalrymple‐Alford is employed by the University of Canterbury. He received research support from the University of Canterbury, Canterbury Medical Research Foundation, Health Research Council of New Zealand, Neurological Foundation of New Zealand, Lotteries Health Research, and Brain Research New Zealand—Rangahau Roro Aotearoa. Tim Anderson is employed by the University of Otago and Anderson Neurology Ltd. He has received funding from the Health Research Council of NZ, and Brain Research New Zealand—Rangahau Roro. Toni Pitcher is employed by the University of Otago. She has received funding from Neurological foundation of NZ, Brain Research NZ—Rangahau Roro Aotearoa and the New Zealand Brain Research Institute. Daniel J. Myall is employed by the New Zealand Brain Research Institute. He received support from the Canterbury Medical Research Foundation, New Zealand Brain Research Institute, and Brain Research New Zealand—Rangahau Roro Aotearoa over the past 12 months.

## Supporting information


**TABLE S1** Supporting information

## Data Availability

Full incidence data, model specification, and analysis code is available at https://github.com/nzbri/parkinsons-multistep.
