# Supplementary material for: A Multi‐Step Model of Parkinson's Disease Pathogenesis
Source: Mov Disord. 2021 Aug 10;36(11):2530–8. doi: 10.1002/mds.28719 (PMC9290013; doi:10.1002/mds.28719)
Supplement: Supplementary file 1 — TABLE S1 Supporting information [file MDS-36-2530-s001.docx]

| **Parameter** | | **Susceptibility model** | **Beta model** | **Armitage-Doll model** |
| --- | --- | --- | --- | --- |
| **k** | Female | 8.0 [7.6, 8.4] | 10.9 [10.1, 11.7] | 6.9 [6.5, 7.4] |
|  | Male | 7.7 [7.5, 7.9] | 10.5 [9.9, 11.1] | 7.1 [6.8, 7.4] |
| **α** | Female | 4.4 [0.5, 16.2] × 10^-6^ | 8.3 [7.9, 8.8] | 4.5 [4.2, 4.9] × 10^-3^ |
|  | Male | 9.9 [3.9, 18.6] × 10^-6^ | 8.5 [8.1, 8.9] | 5.1 [4.8, 5.3] × 10^-3^ |
| **β** | Female | n/a | 1.09 [1.05, 1.12] × 10^-2^ | n/a |
|  | Male | n/a | 1.06 [1.02, 1.09] × 10^-2^ | n/a |
| **C** | Female | 6.4 [6.1, 6.6] | n/a | n/a |
|  | Male | 11.2 [11.0, 11.5] | n/a | n/a |

**Supplementary Table 1.** Parameter estimates from models fitting full age range (30-100+ years). The *Susceptibility model* provided the closest fit of the data. For this model, **k** = number of steps required, $\boldsymbol{\alpha}$ = combined risk of exposure to these steps, $\boldsymbol{∁}$ = Initial percentage of the population susceptible to ever developing Parkinson’s disease. Note that for the *Beta and Armitage-Doll models*, **k** is equal to one less than the required number of steps.
